# Real-World Treatment Patterns, Survival, and Costs for Ovarian Cancer in Canada: A Retrospective Cohort Study Using Provincial Administrative Data

**DOI:** 10.36469/jheor.2021.29145

**Published:** 2021-12-09

**Authors:** Manjusha Hurry, Shazia Hassan, Soo Jin Seung, Ryan N. Walton, Ashlie Elnoursi, Jacob D. McGee

**Affiliations:** 1 AstraZeneca Canada; 2 Sunnybrook Research Institute; 3 London Health Sciences Centre

**Keywords:** ovarian cancer, treatment patterns, survival, costs, resource utilization, administrative data, Canada

## Abstract

**Background:** In 2020, approximately 3100 Canadian women were diagnosed with ovarian cancer (OC), with 1950 women dying of this disease. Prognosis for OC remains poor, with 70% to 75% of cases diagnosed at an advanced stage and an overall 5-year survival of 46%. Current standard of care in Canada involves a combination of cytoreductive surgery and platinum-based chemotherapy.

**Objective:** There are few studies reporting current OC costs. This study sought to determine patient characteristics and costs to the health system for OC in Ontario, Canada.

**Methods:** Women diagnosed with OC in Ontario between 2010 and 2017 were identified. The cohort was linked to provincial administrative databases to capture treatment patterns, survival, and costs. Overall total and mean cost per patient (unadjusted) were reported in 2017 Canadian dollars, using a macro-based costing methodology called GETCOST. It is programmed to determine the costs of short-term and long-term episodes of health-care resources utilized.

**Results:** Of the 2539 OC patients included in the study, the mean age at diagnosis was 60.4±11.35 years. The majority were diagnosed with stage III disease (n=1247). The only treatment required for 74% of stage I patients and 54% of stage II patients was first-line (1L) platinum chemotherapy; in advanced stages (III/IV) 24% and 20%, respectively, did not receive further treatment after 1L therapy. The median overall survival (mOS) for the whole cohort was 5.13 years. Survival was highest in earlier stage disease (mOS not reached in stage I/II), and dropped significantly in advanced stage patients (stage III: mOS=4.09 years; stage IV: mOS=3.47 years). Overall mean costs in patients stage I were CAD $58 099 compared to CAD $124 202 in stage IV.

**Discussion:** The majority of OC patients continue to be diagnosed with advanced disease, which is associated with poor survival and increased treatment costs. Increased awareness and screening could facilitate diagnosis of earlier stage disease and reduce high downstream costs for advanced disease.

**Conclusion:** Advanced OC is associated with poor survival and increased costs, mainly driven by hospitalizations or cancer clinic visits. The introduction of new targeted therapies such as olaparib could impact health system costs, by offsetting higher downstream costs while also improving survival.

## BACKGROUND

It was estimated that in 2020, approximately 3100 Canadian women were diagnosed with ovarian cancer (OC), with approximately 1950 women dying from this disease.^1^ OC is predominantly a disease of older, postmenopausal women, with >80% of cases being diagnosed in women over 50, with the median age of 63 in the overall patient population.^2,3^ Overall, 13% of OC patients are attributable to germline mutations in *BRCA1* or *BRCA2*.^4,5^ Prognosis for OC remains poor, with 70% to 75% of patients being diagnosed at an advanced stage and an overall 5-year survival of 46%.^6^

Most patients who receive first-line (1L) platinum-based chemotherapy with curative intent have no evidence of disease post-treatment. However, tumor recurrence occurs in many patients at a median of 15 months from diagnosis.^2,7^ Current standard of care in Canada involves a combination of cytoreductive surgery and platinum-based chemotherapy.^7–10^ The most common surgery includes removal of the uterus, ovaries and fallopian tubes, omentum, and cytoreduction of peritoneal or retroperitoneal disease.^11^ Cytoreduction to no macroscopic residual disease is the strongest predictor of survival.^12^ Standard adjuvant chemotherapy in OC is often platinum based; however, patients can develop platinum resistance or become platinum refractory.^9^ Patients deemed platinum-sensitive may receive multiple lines of platinum-based therapy. In addition, the duration of response decreases with subsequent treatment courses. In addition, accumulating toxicities, including hypersensitivity reactions, may limit long-term platinum use in patients.^13,14^ Recurrent OC is considered incurable and is associated with limited treatment options.

In Ontario, in patients with a “high risk” of progression, bevacizumab is funded in the frontline setting in combination with paclitaxel and carboplatin or in the recurrent setting in combination with pegylated liposomal doxorubicin or topotecan.^15^ In Canada, olaparib is approved and funded for use as maintenance treatment in platinum- sensitive relapsed OC with the *BRCA* mutation, and approved in April 2019 in Canada for the maintenance treatment of patients with advanced *BRCA*-mutated OC who are in response to 1L platinum chemotherapy, based on the results of the SOLO-1 trial.^16^ Recently, niraparib has been approved and funded as monotherapy for the maintenance treatment of patients with recurrent epithelial ovarian, fallopian tube, or primary peritoneal cancer who are in a complete or partial response to platinum-based chemotherapy, as well in the second-line (2L) setting as maintenance treatment in platinum-sensitive relapsed OC. These poly-adenosine diphosphate–ribose polymerase (PARP) inhibitors trap PARP on DNA at sites of single-strand breaks and prevent their repair, generating double-strand breaks that cannot be repaired accurately in tumors with *BRCA1/BRCA2* or other homologous recombination repair defects.^17^ The use of PARP inhibitors leads to an accumulation of DNA damage and tumor-cell death.^16^

## OBJECTIVES

Given the changing treatment landscape, understanding current outcomes and costs associated with the treatment of patients with OC is important to determine the value of newly approved agents. In Canada, several studies have published survival in OC patients, with some focusing mainly on patients with the BRCA mutation.^12,18–20^ Given the limited published literature currently available, our study set out to characterize treatment patterns, outcomes, and costs in OC patients who have received surgery in the Canadian province of Ontario.

## METHODS

### Study Design

A retrospective cohort study was conducted in women diagnosed with OC between April 1, 2010, and March 31, 2017, with follow-up data until March 31, 2018, identified in the Ontario Cancer Registry using relevant International Classification of Diseases for Oncology version 3 code. Ontario has a population of 14 million residents and provides publicly funded health-care services through the Ontario Health Insurance Plan (OHIP).

### Patient Population

Included patients had to be at least 18 years of age with valid provincial coverage and diagnosed with OC based on the following histology codes from the International Classification of Diseases for Oncology version 3: C48.x-malignant neoplasm of retroperitoneum and peritoneum and C56.x- malignant neoplasm of ovary; received a bilateral salpingo-oophorectomy with or without hysterectomy within the first year of diagnosis, followed by platinum-based chemotherapy. Women were excluded if they had any prior cancer diagnosis.

### Data Sources

Since 1992, the Ontario government has allowed the Institute of Clinical Evaluative Sciences (ICES) to secure and analyze its residents’ health information via administrative databases with ICES scientists and collaborators. Since 2016, ICES Data & Analytic Services has been providing analytic services to non-ICES researchers in the private sector, as was the case for this study. To determine the trajectory of care over time of a patient cohort, health information on each individual patient was linked to applicable datasets. For patients with ovarian cancer, linkages were made to the following data sets: Activity Level Reporting, Hospital Discharge Abstract Database, National Ambulatory Care Reporting System, New Drug Funding Program (NDFP), Ontario Cancer Registry, Ontario Drug Benefit Claims (ODB), OHIP Physician and Laboratory Services, Same Day Surgery, Registered Persons Database, and other datasets that included Continuing Care Reporting System, *Dialysis* Measurement, Analysis and Reporting system and National Rehabilitation Reporting System. The Registered Persons Database contains demographic information on all individuals with OHIP coverage (eg, date of birth, date of death), the Discharge Abstract Database has information about inpatient hospitalizations, and the National Ambulatory Care Reporting System reports the number of cancer clinic, emergency department, and other outpatient clinic visits based on visit dates at any health-care facility in Ontario. The OHIP database captures physician visits and fees for health professionals including general practitioners and medical oncologists while the ODB database includes all oral medications, molecular targeted therapies, and a wide range of supportive care drugs (eg, analgesics and antiemetics) in patients aged ≥65 years or patients on social assistance. The NDFP captures intravenous systemic chemotherapy agents that are publicly funded by Cancer Care Ontario. If treatment information was not available in either ODB or NDFP, treatment information from the Activity Level Reporting database was used. Lastly, the Same Day Surgery database reported on same day surgical procedures.

### Statistical and Costing Analysis

Statistical analyses were performed in SAS Enterprise Guide 7.1. Patient demographics are summarized by number and percentage for categorical variables and by mean and standard deviation for continuous variables, stratified by stage of disease (including “missing”). Staging of disease at ICES is reported using a collaborative staging methodology^21^ and reported only once at the time of diagnosis. Treatment lines and patterns are reported post-surgery and are characterized by the number and percentage of patients receiving different types of treatments (Figure 1). Clinical outcome of interest was overall survival, defined from the time of diagnosis to date of death for known deaths reported in the Registered Persons Database (for mortality), or assumed to be alive if there was a date of last date recorded of health-care encounter in Ontario using a Kaplan-Meier analysis based on stratification and log-rank test (Figure 2). As the number of treatment cycles could not be explored, treatment duration (in months) was reported instead, based on start and stop dates of each treatment. Time between lines of treatment was explored as a proxy for disease progression. A change in line of treatment was noted if there was a gap of at least one month after the last date of the last cycle of the previous line.

**Figure:**
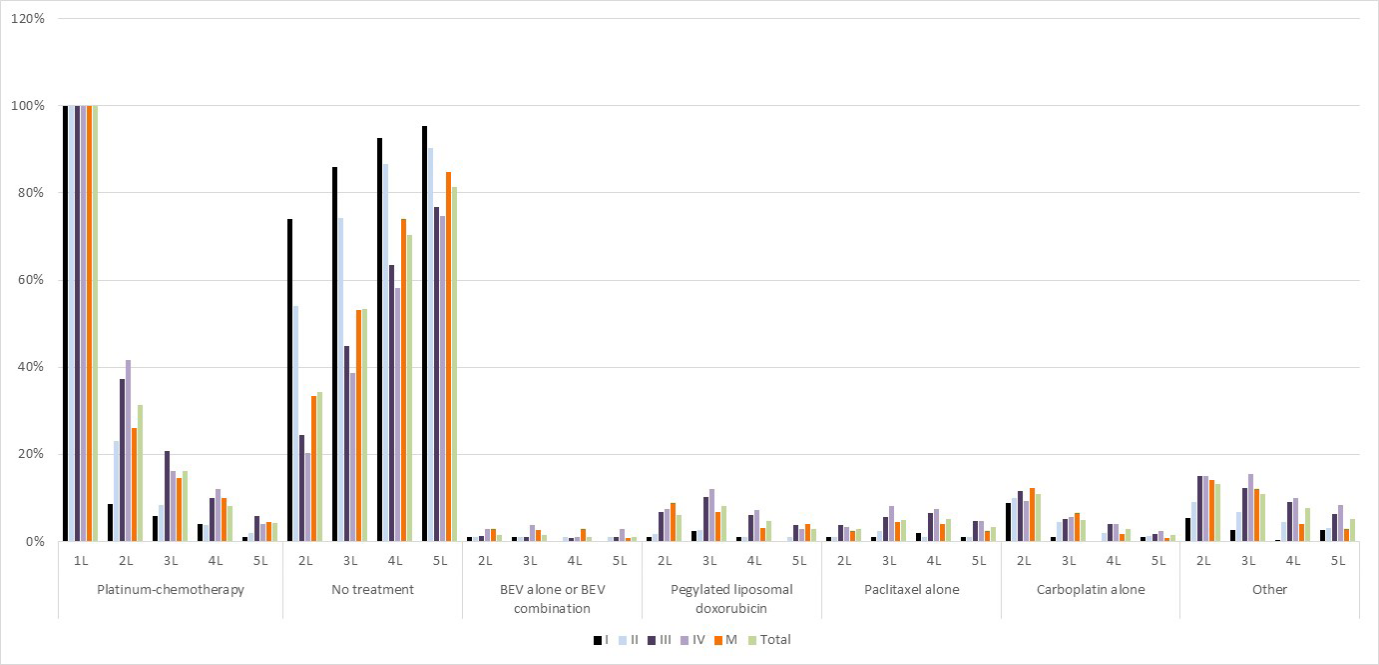
Figure 1. Treatment Patterns Stratified by Stage of Disease

**Figure:**
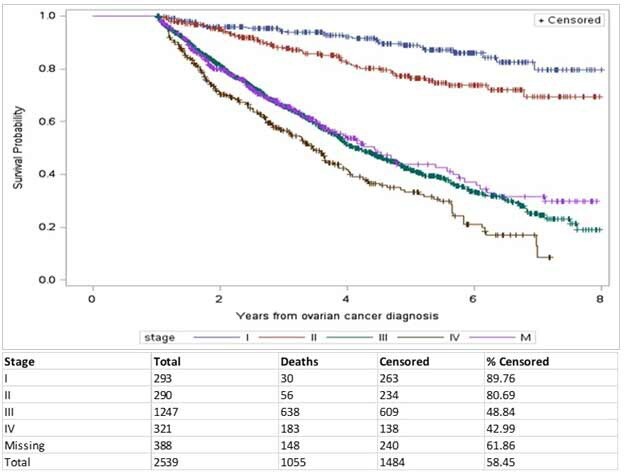
Figure 2. Kaplan Meier Curve of Overall Survival, Stratified by Stage of Disease

A resource use and analysis of direct medical costs (unadjusted) was undertaken to understand the current spending associated with patients with OC in Ontario. Overall total and mean cost per patient is reported in 2017 Canadian dollars, using a macro-based costing methodology, GETCOST, that is available from ICES.^22^ The ICES GETCOST macro was used to determine resource utilization and the total direct medical costs for this cohort. This costing methodology has been described in a previous publication.^23^

To determine costs attributable to OC, the cohort of women with OC (“cases”) were matched to individuals from the general population in Ontario (“controls”). Patients were matched 1:5 on age within 1 year, sex (female), location of health network, location of residence (urban or rural), income quintile, and Charlson comorbidity score for inpatient diagnosis on the basis of sociodemographics and previously described in another study.^24^ OC-attributable costs and resources were calculated by subtracting costs of the controls from the cases.

### Ethics Review

This study was approved by the Sunnybrook Health Sciences Centre Research Ethics Board (REB #317-2017).

## RESULTS

A total of 6221 women were diagnosed with OC during the study period, of whom 2539 were included for analysis. Women were excluded (n=3682) for having no surgery reported within 1 year of diagnosis, no 1L platinum therapy, other prior cancer diagnosis (in order to ensure OC was newly diagnosed), non-residency or less than 1 year follow-up. Mean age at diagnosis was 60.4 years (±11.35), with the majority diagnosed with stage III disease (n=1247). Nearly 70% of patients with stage III or IV were diagnosed at ≥55. The majority reported the location of cancer to be in the ovary (96.3%) with the majority having a histology of epithelial tumors (93.2%). Various surgical approaches were reported, with ovarian debulking being reported in 42.9% of patients (Table 1). Additional patient characteristics are reported in **Tables S1 and S2** in the **Online Supplementary Material**.

**Table 1. Baseline Characteristics Stratified by Stage:** 

	**Stage 1**	**Stage 2**	**Stage 3**	**Stage 4**	**Missing Stage**	**Total**
	**n=293**	**n=290**	**n=1247**	**n=321**	**n=388**	**n=2539**
**Age**						
Mean (standard deviation)	56.33 ± 11.70	59.18 ± 11.44	61.34 ± 11.01	60.97 ± 11.50	60.65 ± 11.27	60.36 ± 11.35
Median (IQR)	56 (49-63)	58 (51-67)	62 (54-69)	62 (52-70)	61 (53-69)	61 (52-69)
Age group						
18-45	52 (17.7%)	32 (11.0%)	97 (7.8%)	27 (8.4%)	33 (8.5%)	241 (9.5%)
46-54	83 (28.3%)	78 (26.9%)	250 (20.0%)	73 (22.7%)	81 (20.9%)	565 (22.3%)
55-64	92 (31.4%)	90 (31.0%)	393 (31.5%)	85 (26.5%)	129 (33.2%)	789 (31.1%)
65-74	43 (14.7%)	55 (19.0%)	371 (29.8%)	92 (28.7%)	109 (28.1%)	670 (26.4%)
75+	23 (7.8%)	35 (12.1%)	136 (10.9%)	44 (13.7%)	36 (9.3%)	274 (10.8%)
**Rurality**						
Urban	256 (87.4%)	245 (84.5%)	1086 (87.1%)	289 (90.0%)	356 (91.8%)	2232 (87.9%)
Rural	37 (12.6%)	45 (15.5%)	161 (12.9%)	32 (10.0%)	32 (8.2%)	307 (12.1%)
**Income quintile**						
Missing	*1 – 5	0 (0.0%)	*1 - 5	0 (0.0%)	*1 - 5	*1 - 5
1 (lowest)	*38 - 42	42 (14.5%)	*193 - 197	59 (18.4%)	*63 - 67	*405 - 409
2	61 (20.8%)	63 (21.7%)	258 (20.7%)	58 (18.1%)	78 (20.1%)	518 (20.4%)
3	54 (18.4%)	54 (18.6%)	256 (20.5%)	51 (15.9%)	69 (17.8%)	484 (19.1%)
4	71 (24.2%)	67 (23.1%)	238 (19.1%)	73 (22.7%)	92 (23.7%)	541 (21.3%)
5 (highest)	64 (21.8%)	64 (22.1%)	297 (23.8%)	80 (24.9%)	81 (20.9%)	586 (23.1%)
**Charlson Comorbidity Index (inpatient diagnoses)**
**Mean (standard deviation)**	0.08 ± 0.28	0.05 ± 0.26	0.11 ± 0.40	0.11 ± 0.38	0.12 ± 0.37	0.10 ± 0.37
**Charlson category**						
0 (lowest)	271 (92.5%)	278 (95.9%)	1143 (91.7%)	292 (91.0%)	347 (89.4%)	2331 (91.8%)
1	*17 - 21	*7 - 11	83 (6.7%)	*24 - 28	35 (9.0%)	174 (6.9%)
2+ (highest)	*1 - 5	*1 - 5	21 (1.7%)	*1 - 5	6 (1.5%)	34 (1.3%)
**Surgery type**						
Missing	*1 - 5	*2 - 6	15 (1.2%)	*1 - 5	*2 - 6	33 (1.3%)
Hysterectomy with omentectomy for malignancy	88 (30.0%)	70 (24.1%)	203 (16.3%)	50 (15.6%)	61 (15.7%)	472 (18.6%)
Ovarian debulking for carcinoma of stage 2C, 3B, 3C, or 4	36 (12.3%)	86 (29.7%)	616 (49.4%)	167 (52.0%)	184 (47.4%)	1089 (42.9%)
Oophorectomy and/or oophorcystectomy	33 (11.3%)	30 (10.3%)	31 (2.5%)	6 (1.9%)	19 (4.9%)	119 (4.7%)
Abdominal/vaginal hysterectomy	88 (30.0%)	57 (19.7%)	140 (11.2%)	36 (11.2%)	68 (17.5%)	389 (15.3%)
Radical (Wertheim) hysterectomy	25 (8.5%)	26 (9.0%)	190 (15.2%)	47 (14.6%)	38 (9.8%)	326 (12.8%)
Oophorectomy with total omentectomy	*18 - 22	*15 - 19	52 (4.2%)	*10 - 14	*12 - 16	111 (4.4%)

*Exact counts suppressed due to privacy reasons.

### Treatment Patterns

The meantime from diagnosis to the start of systemic treatment irrespective of stage was 3.14±7.22 months, likely accounting for recovery following cytoreduction. In the overall cohort, all patients received 1L platinum-based either in combination or as monotherapy. Upon completion of 1L treatment, 74% of stage I and 54% of stage II patients did not receive subsequent treatment. However, in patients with stage III and IV disease, only 24% and 20% did not receive any subsequent treatment, respectively. More stage IV patients received platinum-based therapies in 2L while more stage III patients received this treatment in third-line (3L). After platinum-combination chemotherapy, the use of pegylated liposomal doxorubicin, single-agent carboplatin, or single-agent paclitaxel were more frequently used in subsequently lines of treatment, with very limited use of bevacizumab (Figure 1).

Although mean time (in months) on treatment was not determined by type of treatment, it was determined for each treatment line and generally decreased with each additional treatment line: 3.93±2.14 months for all 1L treatments, 2.94±3.23 months for all 2L treatments and 3.11±4.03 months for all 3L treatments.

### Outcomes

The median overall survival (mOS) for the whole cohort was 5.13 years (95% CI 4.80-5.57). Survival was highest in the earlier stage of disease, with the mOS not reached in stage I or II. Survival dropped significantly in patients with advanced disease, with mOS of 4.09 years (95% CI 3.92-4.48) for stage III and 3.47 years (95% CI 3.09-3.79) for stage IV. Patients missing staging at diagnosis had a mOS of 4.44 years (95% CI 3.73- 5.57). Log rank test was used to compare survival between the stages and statistical significance was found for stage I to III, I to IV, II to III and II to IV (*P*<0.0001). Five-year survival rates by stage were: stage I=89%, stage II=76%, stage III=42%, stage IV=33%, and missing stage=44% (Figure 2).

In patients who received subsequent lines of treatment, time between lines of treatment (“time to first subsequent treatment”) was further explored, as a proxy for “progression-free survival.” On average, “time to first subsequent treatment” from 1L to 2L systemic chemotherapy was similar in stage I or stage II disease: 13.5 (±19.8) months and 14.9 (±17.1) months, respectively. As expected, in patients with stage III or IV disease, “time to first subsequent treatment” was shorter than earlier stages, with a mean estimate of 11.2 (±12.0) and 8.7 (±10.0) months, respectively (Figure 3).

**Figure:**
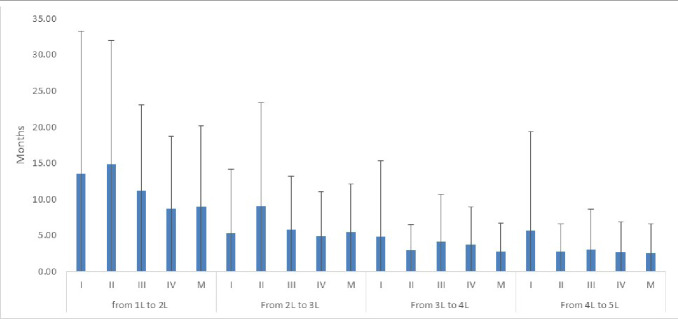
Figure 3. Time Between Lines of Treatment Stratified by Stage of Disease

### Costs and Resources

For the OC (“cases”) cohort (n=2539), the overall total cost during the study period was CAD $258M, with a mean cost per patient of CAD $101 707±69 997. For the matched controls (n=12 695), the overall total cost was CAD $504M, with a mean cost per patient of CAD $39 693± CAD $89 396, resulting in a mean cost per patient of CAD $62 014 attributable to OC. Cost differences between the two cohorts were the greatest for inpatient hospitalization, cancer clinic visits, OHIP specialist visits and IV chemotherapy (see **Tables S3A and S3B** in the **Online Supplementary Material**).

Figure 4 presents costs of the OC cohort stratified by stage and by resource (since differentials with controls could not be determined, as controls could not be matched by stage). Mean cost per patient per stage was I=CAD $58 099± CAD $56 004; II=CAD $71 445± CAD $53 214; III=CAD $114 713± CAD $70 649; IV=CAD $124 202± CAD $71 136. Advanced stages had almost double the mean cost per patient for cancer clinic visits and inpatient hospitalizations compared to early stages.

**Figure:**
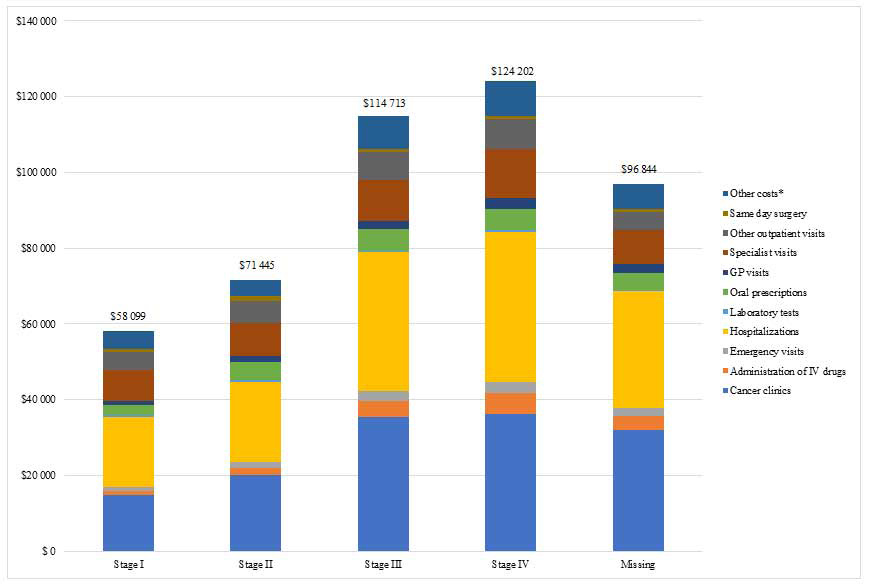
Figure 4. Mean Cost per Patient, Stratified by Stage of Disease *Other includes costs for complex continuing care, dialysis, non-physician costs, shadow billing and rehabilitation.

## DISCUSSION

This retrospective, population-level study identified a cohort of 2539 women diagnosed with OC in Ontario, Canada, between 2010 and 2017. The objective of this study was to understand the treatment approaches, outcomes, and costs associated with OC in patients having received surgery and treated with platinum-chemotherapy. Compared to previous studies reported in Ontario, Manitoba, British Columbia, and the United Kingdom, age and stage distribution at diagnosis were comparable, with stage III being diagnosed most frequently.^12,18–20,25,26^ Although our study included the most recent cohort of patients diagnosed with OC, limited changes in stage distribution over time suggest a lack of early detection methods for epithelial ovarian cancer, resulting in poor survival.^27^ Platinum-chemotherapy was used most frequently, albeit in only 16% to 31% of patients in subsequent lines. The low use of bevacizumab is likely reflective of the funding criteria in Ontario; the criteria allow the use of bevacizumab in patients with high risk of relapse defined as stage III patients who are suboptimally debulked, or stage III unresectable, or stage IV patients). Our study included only resected patients. Variation in treatment approach can be explained by clinical equipoise, where physicians tailor treatment regimens at recurrence based upon patient symptomatology, sensitivity to platinum-chemotherapy, anticipated side effects, as well as the availability of funding for a treatment regimen. As well, there is a lack of central direction from Cancer Care Ontario/provincial treatment funders regarding treatment best practice. The low use of subsequent bevacizumab is likely a reflection of the funding criteria for bevacizumab in Ontario, being restricted to patients with high risk of progression.^15^

The direct medical costs attributable to OC were determined by comparing overall and mean cost per patient for the overall OC cohort and matched controls (**Tables S3A and S3B** in the **Online Supplementary Material**). Then, costs for the overall OC cohort were stratified by disease stage and calculated from a provincial perspective (Figure 4) Overall mean costs in stage I OC patients compared to stage IV were CAD $58 099 and CAD $124 202, respectively. Cost drivers for both stages were inpatient hospitalizations and cancer clinic visits; however, costs associated with chemotherapy may be underestimated. Although our study was not able to track early-stage OC patients who progressed or patients who relapsed, a recent study of patients with relapsed stage III/IV OC reported a mean cost of CAD $52 227 (2016), with in-hospital care accounting for 71% and chemotherapy for 17% of total costs.^28^ Another study in Ontario reported phase-specific costs over a study period of 1997-2007, with a mean per patient cost of OC one year before diagnosis of CAD $2098 (2009) and CAD $29 640 in patients who survived beyond the first year, compared to CAD $46 270 in patients who died during the first year.^29^ Comparing our estimated cost to a similar public health care setting in Australia, the mean cost of early OC (stage I or II) was AUD $31 958 compared to AUD $50 945 (2008 costs) in advanced disease (stage III and IV), with a patient follow-up of 2.5 years.^30^ The cost driver in Australia was mainly chemotherapy (44%) followed by surgical treatment (32%).

### Limitations

This study does have limitations: (1) the lack of available disease characteristics (eg, *BRCA* mutations) are not captured in the ICES data, which would enable specific interpretation of clinical outcomes; (2) collaborative staging at diagnosis was used and assumed to be closely related to the International Federation of Gynecology and Obstetrics criteria, although some patients could be misclassified as a result; (3) surgical outcomes such as status of residual disease or complications related to surgery were not assessed; (4) we are unable to determine whether patients remain progression-free or cured, which can partly explain the high number of patients who do not receive subsequent treatment in stage I or II disease; (5) the public drug coverage database does not capture patients who are under the age of 65, unless they are on the Trillium Drug Program or receive medication through the Exceptional Access Program; (6) we estimated that the impact of using drug costs from the ODB and NDFP on overall costs to be minimal, as the Activity Level Reporting database generally captures the “cheaper” intravenous chemotherapies: those drug costs have not been included in the costing algorithm, while the NDFP does include the drug costs of the more expensive intravenous chemotherapies; and (7) utilization of newer therapies are not captured in this analysis, ie, olaparib, as it was not funded in the province at the time of analysis.

## CONCLUSION

Advanced OC is associated with poor survival and increased costs, mainly driven by hospitalizations or cancer clinic visits. The majority of OC patients in our study were diagnosed with stage III or IV disease at diagnosis. The introduction of new targeted therapies, such as olaparib based on the results of the SOLO-1 trial, could impact health system costs, especially those associated with advanced disease, by offsetting higher downstream costs such as costly hospitalizations, while also improving survival.

### Conflicts of Interest

SJS, MH, RNW, and AE are all employees of AstraZeneca; SH and SJS received an unrestricted grant from AstraZeneca Canada Inc. to conduct this study; JDM reported personal fees from AstraZeneca during the conduct of the study, and personal fees from AstraZeneca and Merck outside the submitted work.

### Author Contributions

All authors (MH, SH, SJS, RNW, AE, JDM) met the following criteria: substantial contributions to the conception or design of the work; or the acquisition, analysis, or interpretation of data for the work; drafting the work or revising it critically for important intellectual content; final approval of the version to be published; and agreement to be accountable for all aspects of the work in ensuring that questions related to the accuracy or integrity of any part of the work are appropriately investigated and resolved.

## Figures and Tables

**Figure:** Online Supplementary Material Tables S1, S2, and S3A and B.
